# A Polarization-Insensitive, Vanadium Dioxide-Based Dynamically Tunable Multiband Terahertz Metamaterial Absorber

**DOI:** 10.3390/ma17081757

**Published:** 2024-04-11

**Authors:** Mohsin Raza, Xiaoman Li, Chenlu Mao, Fenghua Liu, Hongbo He, Weiping Wu

**Affiliations:** 1 Laboratory of Thin Film Optics, Shanghai Institute of Optics and Fine Mechanics, Chinese Academy of Sciences, 390 Qinghe Road, Jiading District, Shanghai 201800, China; razamohsin@siom.ac.cn (M.R.); lixiaoman@siom.ac.cn (X.L.); maochenlu@siom.ac.cn (C.M.); fenghualiu@siom.ac.cn (F.L.); 2 State Key Laboratory of High Field Laser Physics, Shanghai Institute of Optics and Fine Mechanics, Chinese Academy of Sciences, 390 Qinghe Road, Jiading District, Shanghai 201800, China; 3 University of Chinese Academy of Sciences, Beijing 100049, China

**Keywords:** metasurface, perfect absorber, terahertz functional device, six-band absorption, terahertz radiation

## Abstract

A tunable multiband terahertz metamaterial absorber, based on vanadium dioxide (VO_2_), is demonstrated. The absorber comprises a three-layer metal–insulator–metal (MIM) configuration with a split ring and slots of VO_2_ on the uppermost layer, a middle dielectric substrate based on silicon dioxide (SiO_2_), and a gold reflector on the back. The simulation results indicate that, when VO_2_ is in the metallic state, the proposed metamaterial exhibits nearly perfect absorption at six distinct frequencies. The design achieves an average absorption of 98.2%. The absorptivity of the metamaterial can be dynamically tuned from 4% to 100% by varying the temperature-controlled conductivity of VO_2_. The proposed metamaterial absorber exhibits the advantages of polarization insensitivity and maintains its absorption over 80% under different incident angle conditions. The underlying physical mechanism of absorption is explained through impedance matching theory, interference theory, and the distribution of electric fields. The ability to achieve multiband absorption with tunable characteristics makes the proposed absorber a promising candidate for applications in terahertz sensing, imaging, communication, and detection. The polarization insensitivity further enhances its practicality in various scenarios, allowing for versatile and reliable performance in terahertz systems.

## 1. Introduction

A terahertz (THz) wave is situated in the frequency range that falls between microwaves and infrared radiation, with a corresponding frequency range of 0.1 to 10 THz. This frequency range holds considerable promise for a diverse range of applications, including, but not limited to communication systems [1], modulators [2], imaging, sensing [3,4,5], and spectroscopy applications [6]. However, because there is a lack of natural materials that respond to THz radiation, this frequency range is the least developed among all. Nonetheless, recent progress in metamaterials has paved the way for the realization of THz functional devices [7,8,9]. Metamaterials exhibit unique electromagnetic responses, making them promising candidates for a wide range of practical applications, including absorbers [10,11], antennas [12], filters, and switches [13,14,15]. THz metamaterial absorbers (MMAs) are important in diverse applications, typically structured with metal, insulator, and metal layers [16,17]. Efficiently inducing resonance at adjacent frequencies yields dual-band [18], multi-band [19], or broadband absorption [20]. Stacking multi-layer structures is an alternative approach for designing absorbers that can achieve multi-band resonances [21]. However, these designs lack tunability except for modifying the resonator size.

Recent research has explored the integration of phase change materials (PCMs) [22,23], such as liquid crystal [24,25], graphene [26,27,28,29], and vanadium dioxide (VO_2_), with MMAs to exploit their active tunability [30,31,32,33,34]. VO_2_ holds significant prominence as a widely used PCM due to its unique properties. Its phase transition can be controlled electronically, thermally, and optically [30], leading to conductivity changes up to five orders of magnitude. VO_2_ remains non-conductive at room temperature until it reaches its critical transition temperature of 340 K [31,35,36]. This transition involves a structural lattice transformation from monoclinic rutile to tetragonal rutile, resulting in a shift from a non-conductive to a conductive phase. This integration enables active tunability, offering precise control over metamaterial absorption characteristics [32].

Notably, researchers have presented innovative VO_2_-based absorbers [33,37,38,39]. Nevertheless, existing THz absorber designs often exhibit narrowband absorption, lack continuous multi-peak tunability, and have complex structures, limiting their functionality. Therefore, a pressing need exists for absorbers with dynamic multi-peak tunability, polarization insensitivity, and a broader absorption bandwidth across a wide frequency range. In this paper, we address these issues with a novel tunable THz metamaterial multiband absorber. Our proposed absorber offers several innovative features that distinguish it from previously developed absorbers. Firstly, it incorporates a unique design that enables multiband absorption by utilizing multiple resonators and tailored geometries. This design approach allows for the achievement of multiple absorption peaks across a wide frequency span, resulting in a significantly higher absorption bandwidth and enhanced functionality. Additionally, our proposed absorber possesses dynamically tunable characteristics, providing precise control of absorption peaks at specific frequencies. Moreover, our absorber exhibits polarization insensitivity, ensuring consistent and reliable absorption performance regardless of the incident wave’s polarization state. The detailed comparison is shown in Table 1. These abilities of the proposed metamaterial absorber expand the practical applications in terahertz sensing, imaging, communication, and other relevant fields.

## 2. Materials and Methods

**Figure 1:**
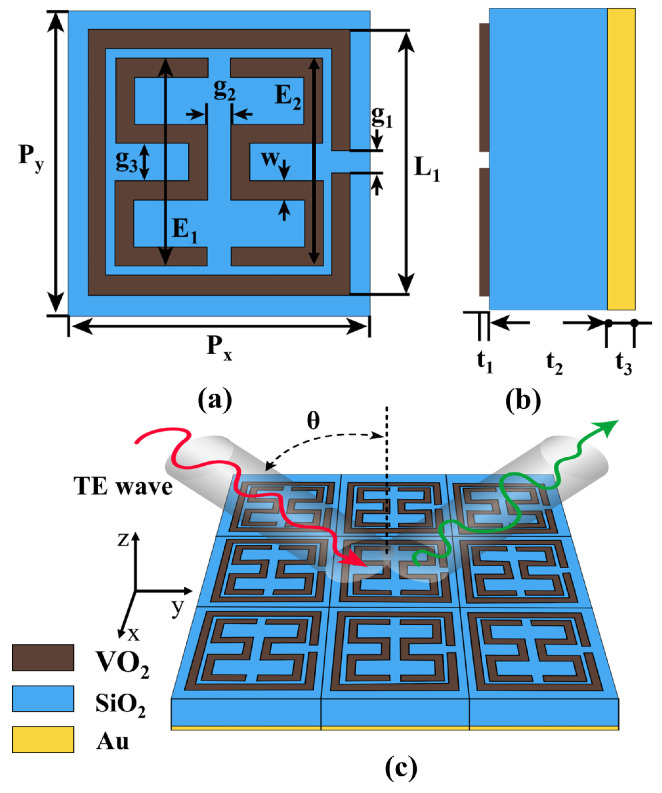
Schematic illustration and design configuration of the proposed MMA: (**a**) The unit cell schematic of the proposed MMA along with its geometric parameters. (**b**) Side view of the unit cell. (**c**) The periodic array arrangement of the proposed MMA, where θ is the incident angle of the EM THz waves, red and green arrows represent incident and reflected THz waves respectively.

**Figure 2:**
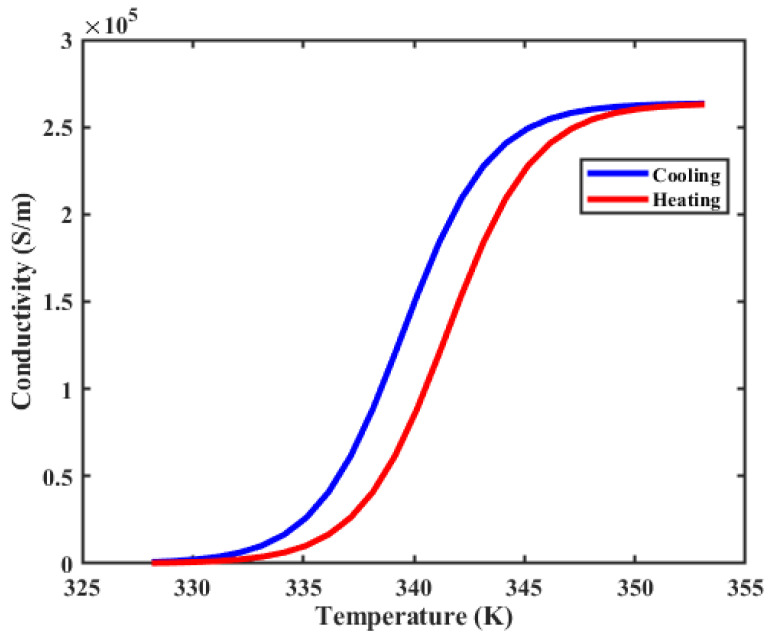
Temperature-dependent conductivity of VO_2_.

The designed unit-cell structure of the MMA is illustrated in Figure 1. It comprises a three-layer configuration consisting of top, VO_2_ resonators, a silicon dioxide (SiO_2_) substrate, and a continuous gold reflector. The optimized geometrical parameters of the proposed metamaterial are as follows: periodicity $P_{x}=P_{y}=80$ µm, length of the outer split ring resonator $L_{1}=70$ µm, and wire width w=5 µm. The lengths of the inner VO_2_ patches are $E_{1}=E_{2}=55$ µm; the gap between the arms of the inner VO_2_ resonators is $g_{2}=6$ µm; the gap of the split ring resonator is $g_{1}=6$ µm, $g_{3}=10$ µm; the thickness of VO_2_ is $t_{1}=0.2$ µm. The SiO_2_ substrate has a thickness of $t_{2}=29$ µm with a dielectric constant of 3.8, which exhibits negligible loss at THz frequencies [47]. The thickness of the gold reflector is $t_{3}=0.2$ µm, with a conductivity of $4.09\;\times \;10^{7}$ S/m within the THz range [48]. In our proposed absorber design, the temperature (T)-dependent conductivity of VO_2_ is calculated by Bruggeman effective medium theory (EMT) [49]. Figure 2 demonstrates the variations in VO_2_ conductivity across temperature changes during both heating and cooling processes, with cooling consistently trailing by 2 K compared to heating. The transition of VO_2_ from the insulating to the metallic states reveals evident hysteresis. The conductivity spans from 200 S/m to $2.5\times 10^{5}$ S/m as the temperature varies between 326 K and 350 K, aligning with the findings from earlier experimental results [50]. We assumed that the variable conductivity of VO_2_ mimics the phase transition effect. The optical permittivity of VO_2_ in the THz range is modeled using the Drude model [48].

(1) $$\epsilon \left(\omega \right)=\epsilon_{\infty }-\frac{\omega_{p}^{2}\left(\sigma \right)}{\omega^{2}+i\gamma \omega }$$

The dielectric permittivity at high frequency is denoted as $\epsilon_{\infty }=12$; γ is the collision frequency with value $\gamma =5.75\times 10^{13}$ rad/s, and $\omega_{p}$ is the conductivity (σ)-dependent plasma frequency, as described by

(2) $$\omega_{p}^{2}\left(\sigma \right)=\frac{\sigma }{\sigma_{0}}\omega_{p}^{2}\left(\sigma_{0}\right)$$

where $\sigma_{0}=3\times 10^{5}$ S/m and $\omega_{p}\left(\sigma_{0}\right)=1.4\times 10^{15}$ rad/s. In the designed structure, the thickness of the gold is larger than the skin depth, leading to the complete reflection of the incident wave and resulting in zero transmittance (T(ω)). Consequently, the absorptance A(ω) can be calculated using the equation

(3) $$A\left(\omega \right)=1-R\left(\omega \right)=1-|S_{11}{\left(\omega \right)|}^{2}\;$$

where R(ω) represents the reflectance and $S_{11}\left(\omega \right)$ is the reflection coefficient in the simulated S-parameters. For the simulations, the S-parameter was obtained using a frequency domain solver implemented in the commercial software *CST Microwave Studio* 2019. The unit cell boundary conditions were specified for the x and y directions, while an open (add space) boundary condition was applied for the z direction. To ensure accurate simulation results, a refined mesh was carefully chosen to achieve high precision.

## 3. Results and Discussion

**Figure 3:**
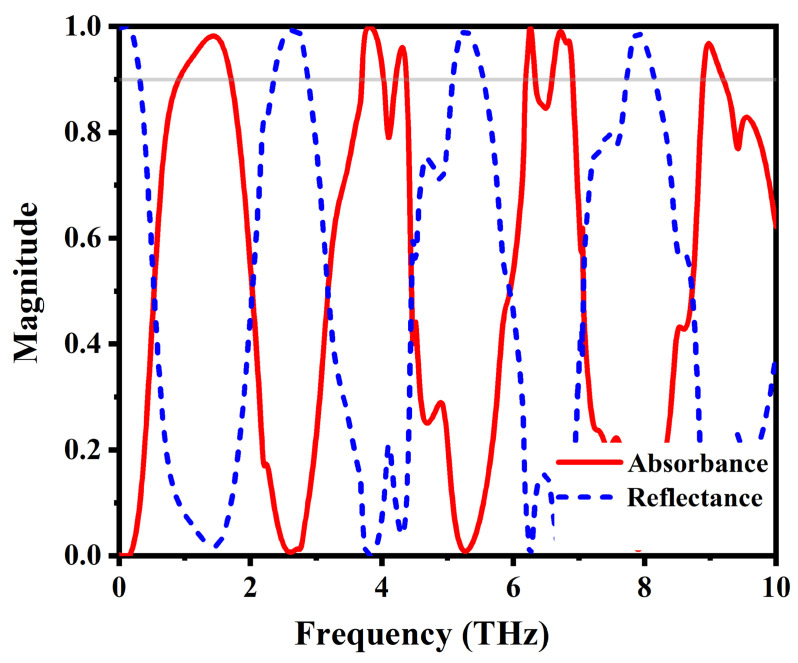
The absorption and reflection spectra of the proposed absorber, when subjected to normal incident conditions while the VO_2_ is in the conducting state. The grey line represents the 90% absorption magnitude.

We analyzed the behavior and functionality of the proposed MMA by subjecting it to excitation within the terahertz spectrum under normal transverse electric (TE) incidents. At T = 344 K, the absorption spectrum, illustrated in Figure 3 with VO_2_ in the conducting state ($2\times 10^{5}$ S/m), reveals six distinct peaks with absorptances exceeding 90%. The proposed design exhibits perfect absorption characteristics at $f_{1}$ = 1.44 THz, $f_{2}$ = 3.81 THz, $f_{3}$ = 4.32 THz, $f_{4}$ = 6.25 THz, $f_{5}$ = 6.77 THz, and $f_{6}$ = 9.03 THz, corresponding to absorptances of 98.2%, 99.9%, 96%, 99.7%, 99%, and 96.4%, respectively. Through the incorporation of VO_2_-based multiple resonators, this configuration attains an average absorption rate of 98.2%, with each band having an absorption bandwidth of 0.78 THz, 0.35 THz, 0.16 THz, 0.15 THz, 0.32 THz, and 0.24 THz, respectively. The quality factor is defined as $Q=\frac{f_{0}}{\mathrm{FWHM}}$, where FWHM is the full-width at half-maximum of the resonant peaks and $f_{0}$ is the resonant frequency. As shown in Figure 3, the resonance $f_{2}$ overlaps with $f_{3}$ and $f_{4}$ with $f_{5}$. To calculate the FWHM, the overlapped curve is treated as a superposition of Lorenz curves. The quality factors for the resonance peaks are 0.96, 3, 7, 11.3, 9, and 11, respectively.

**Figure 4:**
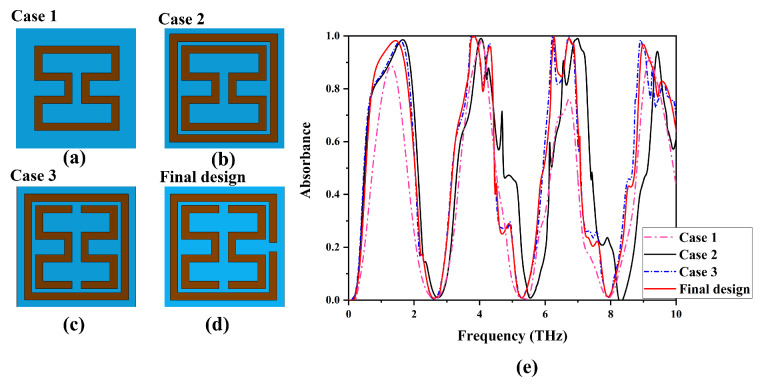
Design steps of proposed MMA with absorption spectra. (**a**) Dumbbell-shaped square rings; (**b**) dumbbell-shaped square rings with outer square ring; (**c**) dumbbell-shaped square rings split as $E_{1}$ and $E_{2}$; (**d**) final design with outer split ring and inner $E_{1}$ and $E_{2}$ resonators; (**e**) absorption spectra of all cases.

During the design process, we explored different geometries for the metamaterial absorber, as depicted in Figure 4. All the parameters are the same as defined in Figure 1a. Initially, we investigated the absorption characteristics of dumbbell-shaped square rings, as illustrated in Figure 4a. The simulation results in Figure 4e reveal four absorption peaks, with two peaks surpassing 90% at 9.01 THz (91%) and the highest absorption reaching 98% at 4.06 THz. Subsequently, in Case 2, considering the square ring positioned outside the dumbbell square ring in Figure 4b, we observe five absorption peaks with over 90% absorption, achieving perfect absorption at 1.66 THz (98%), 4.06 THz (98%), 6.55 THz (90%), 6.98 THz (99%), and 9.42 THz (94%). The sixth resonance at 4.27 THz exhibits 88% absorption. This enhanced absorption is attributed to the closely placed outer ring and inner dumbbell ring, inducing resonance that mutually supports each other. In Case 3, a gap is introduced by cutting the inner dumbbell shape, as shown in Figure 4c. The simulation results demonstrate six absorption peaks with over 98% absorption at 1.56 THz, 3.81 THz, 4.32 THz, 6.25 THz, 6.77 THz, and 9.01 THz. The split of the $E_{1}$ and $E_{2}$ resonators couples with each other to enhance absorption, particularly broadening the 3rd and 4th peaks. Finally, in Case 4, a gap is introduced on the outer ring, as depicted in Figure 4d. A minimal change is observed in the absorption peaks except for the first absorption peak, whose bandwidth increases. This is attributed to the larger effective refractive index of the split ring compared to the ring resonators.

**Figure 5:**
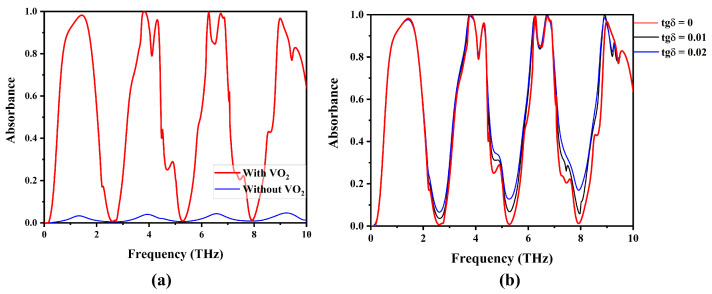
The absorption spectra of the proposed MMA (**a**) with VO_2_ and without VO_2_ (**b**) with tangential losses (tgδ) = 0, 0.01, 0.02.

To explore the impact of dielectric losses, we conducted simulations of the metamaterial absorber with and without VO_2_ resonators, as illustrated in Figure 5. Our findings indicate that the losses in SiO_2_ are below 5%. To further validate this, we simulated the metamaterial absorber with different tangential losses (tgδ) of the dielectric. Figure 5b presents the absorption characteristics with tangential loss values ranging from 0.01 to 0.02. Notably, the tangential loss demonstrated no effect on the first five absorption peaks and exhibited only a negligible influence on the final absorption band. These results reveal that the incorporation of VO_2_ resonators introduces a distinctive design enabling multiband absorption. The primary cause lies in the interplay between the VO_2_ resonator and the underlying continuous gold film, generating the Fabry–Pérot resonance effect. This phenomenon detrimentally affects the incoming THz wave, leading to an enhanced capability to attenuate the wave. This design approach facilitates the attainment of multiple absorption peaks over a broad frequency range, leading to a significantly enhanced absorption bandwidth and improved functionality.

**Figure 6:**
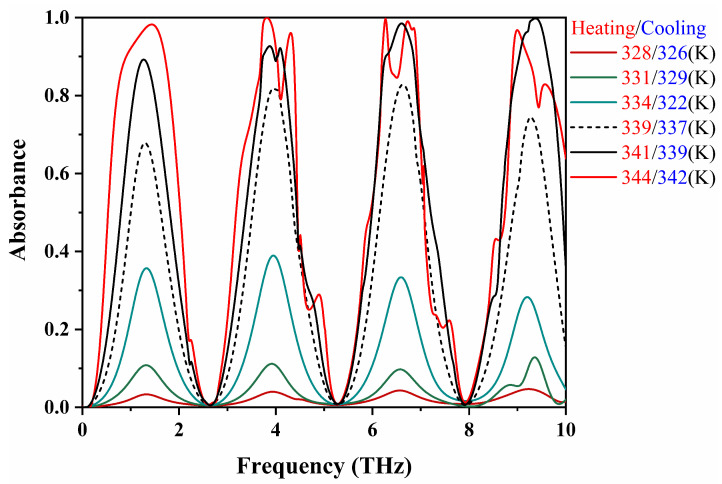
The absorption spectra of the proposed MMA during the heating and cooling of VO_2_ under normal incident conditions.

The modulation of VO_2_’s conductivity with temperature induces variations in its dielectric constant, enabling continuous tuning of absorptivity in our proposed absorber. As depicted in Figure 6, absorptivity across different frequency bands can be continuously adjusted from 4% to 99% by varying VO_2_’s conductivity within the range of 200 S/m to $2\times 10^{5}$ S/m [51,52,53], while the central frequencies remain constant. This study investigates the transition of VO_2_ from its insulated to metallic states, specifically focusing on the heating process occurring within the temperature span of 328 K to 344 K. As illustrated in Figure 6, between the temperatures of 328 K and 334 K, the changes in the absorption rate of the device ranged from 0.4% to 30%. However, around 340 K, there was a substantial increase in the absorption rate, exceeding 70%. Optimal absorption performance was attained at T = 344 K, where the absorption rate reaches a peak value of 0.999, corresponding to a VO_2_ conductivity of $2\times 10^{5}$ S/m. Additionally, at low temperatures, the results reveal the presence of four absorption peaks at 1.26 THz, 3.94 THz, 6.59 THz, and 9.24 THz, respectively, with a consistent frequency interval of 2.65 THz. This phenomenon arises from the Fabry–Pérot resonance occurring within the middle layer. The central frequency interval between adjacent absorption peaks can be computed as $\Delta f=\frac{c_{0}}{2nd\cos\theta }$, where $c_{0}$ represents the velocity of light in vacuum, θ denotes the angle of incidence, $n=(\sqrt{\epsilon })$ represents the refractive index of SiO_2_, and *d* represents the thickness of the dielectric. The calculated frequency interval of 2.65 THz aligns with the simulated results. Moreover, at high temperatures, the conductivity of the VO_2_ resonators increases, leading to the emergence of resonance modes within the outer split ring resonator and the inner arms. The coupling between these modes induces more resonance peaks at 4.32 THz and 6.25 THz.

This controllable and continuous modulation of absorptivity based on VO_2_’s conductivity showcases the absorber’s ability to dynamically adjust its performance across multiple frequency bands, offering a versatile and efficient means of manipulating electromagnetic wave absorption for various applications.

**Figure 7:**
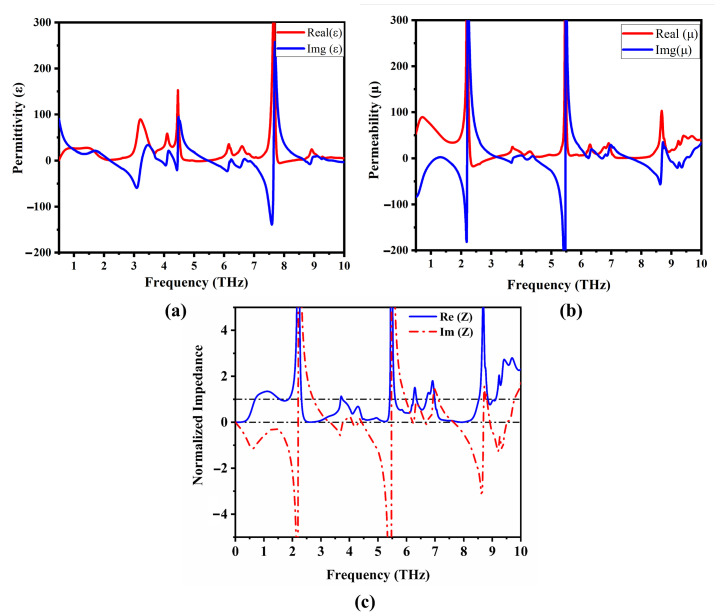
The real and imaginary parts of the effective parameters of the proposed MMA at 344 K with conductivity $2\times 10^{5}$ S/m. (**a**) Effective permittivity; (**b**) effective permeability; (**c**) normalized effective impedance.

The application of effective medium theory justifies the consideration of the designed system as an isotropic uniform medium with effective optical parameters (permittivity, permeability, impedance *Z*) when VO_2_ is in the metallic state [42]. This approach provides a valid and effective method for analyzing the electromagnetic behavior of the system. The effective parameters were extracted using the S-parameter retrieval technique [54]. The effective impedance of the designed absorber can be calculated using the expression

(4) $$\;Z=\sqrt{\frac{\mu }{\varepsilon }}=\sqrt{\frac{{\left(1+S_{11}\right)}^{2}-S_{21}^{2}}{{\left(1-S_{11}\right)}^{2}-S_{21}^{2}}}\;$$

where ε and μ represent the effective permittivity and permeability, respectively. Figure 7a,b show the real and imaginary values of the effective permittivity and permeability, respectively. In the case where the relative permittivity equals the relative permeability, the metamaterial impedance matches the free space impedance (377Ω), resulting in zero reflection. Figure 7c shows the simulated real and imaginary parts of the effective impedance. Notably, the real components of the effective impedance for all absorption peaks approach unity, while the imaginary components approach zero, indicating impedance matching with free space and maximum absorption [55].

**Figure 8:**
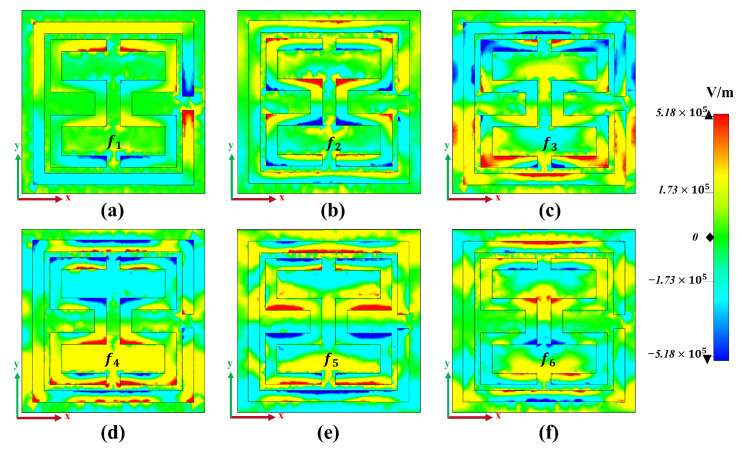
Z-component of the electric field’s real ($E_{Z}$) distributions at resonance frequencies under normal incidence, when VO_2_ is in the conducting state at 344 K. (**a**) Real ($E_{Z}$) at $f_{1}$ = 1.44 THz; (**b**) real ($E_{Z}$) at $f_{2}$ = 3.81 THz; (**c**) real ($E_{Z}$) at $f_{3}$ = 4.32 THz; (**d**) real ($E_{Z}$) at $f_{4}$ = 6.25 THz; (**e**) real ($E_{Z}$) at $f_{5}$ = 6.77 THz; (**f**) real ($E_{Z}$) at $f_{6}$ = 9.03 THz.

**Figure 9:**
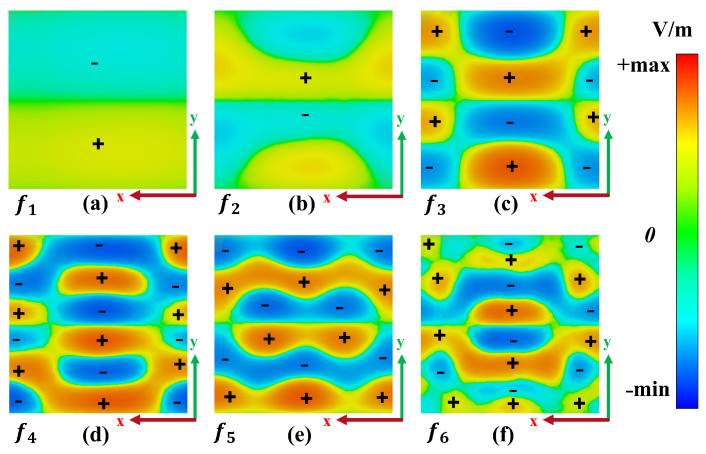
Z-component of the electric field’s real ($E_{Z}$) distributions at the interference of the bottom gold reflector and dielectric substrate at resonance frequencies under normal incident, when VO_2_ is in the conducting state at 344 K. (**a**) Real ($E_{Z}$) at $f_{1}$ = 1.44 THz; (**b**) real ($E_{Z}$) at $f_{2}$ = 3.81 THz; (**c**) real ($E_{Z}$) at $f_{3}$ = 4.32 THz; (**d**) real ($E_{Z}$) at $f_{4}$ = 6.25 THz; (**e**) real ($E_{Z}$) at $f_{5}$ = 6.77 THz; (**f**) real ($E_{Z}$) at $f_{6}$ = 9.03 THz.

To gain a deeper insight into the operational mechanism of the absorber, the analysis focused on the z-component of the electric field’s real ($E_{Z}$) distributions on the proposed metamaterial absorber. Figure 8a and Figure 9a reveal that the electric field density responsible for the initial absorption peak primarily originates from the outer split ring resonator, with opposite charges accumulating at its opening, indicating the excitation of the electric dipole. Figure 8b and Figure 9b demonstrate a shift in the resonant mode and the gradual transfer of electric field strength from the outer split ring to the inner corners of the $E_{1}$ and $E_{2}$ resonators as the incident frequency increases, confirming the excitation of the electric dipole.

The third absorption band in Figure 8c and Figure 9c exhibits a significant electric field distribution on the upper and lower arms of the $E_{1}$ and $E_{2}$ resonators. While the first and second absorption peaks primarily arise from the VO_2_ resonators, the third absorption band exhibits enhanced coupling between the dielectric and surrounding layers [56]. This enhancement leads to the distribution of two dipole pairs with equal and opposite charges across the absorber surface, confirming that the resonance is attributed to the electric quadrupole response of the VO_2_ resonators [57]. This transfer of electric field intensity is also observed for the last three absorption peaks. Figure 8d–f illustrate that the electric field density oscillates within the gaps of the inner $E_{1}$ and $E_{2}$ resonators, as well as the top and bottom gaps between the outer split ring and the inner $E_{1}$ and $E_{2}$ arms. Consequently, the higher absorption observed for these peaks can be attributed to the contributions from the outer split ring and the gaps of the inner $E_{1}$ and $E_{2}$ resonators. Figure 9d,e show that three dipole pairs are induced by the positive and negative charges, forming an electric hexapole mode resonance for both absorption bands at 6.25 THz and 6.77 THz; however, for the last absorption band, the positive and negative charges induce four pairs of dipoles, confirming the excitation of an electric octopole mode resonance. This design not only excites the basic mode, but also the higher mode of resonance, contributing to the formation of multiband absorption.

Figure 9 reveals that the electric field is not concentrated solely at the interference region between the VO_2_ layer and the SiO_2_ dielectric substrate. The VO_2_ layer effectively interacts with the incident waves, and a portion of these waves transmits inside the dielectric spacer, resulting in the formation of a cavity. With the increase in frequency, this cavity leads to constructive interference, giving rise to Fabry–Pérot resonance [42]. Thus, the structure acts as a Fabry–Pérot cavity between the top VO_2_ resonators and the metallic reflector. The Fabry–Pérot absorption phenomenon observed in the proposed design can be effectively explained and interpreted through the application of interference theory [58].

According to the interference theory model, incident plane waves interact with the structure, leading to reflected and transmitted waves. The reflection and transmission coefficients of these plane waves can be represented as ${\tilde{r}}_{12}=r_{12}e^{i\phi_{12}}$, ${\tilde{t}}_{12}=t_{12}e^{i\theta_{12}}$, respectively. Due to the presence of a metallic reflector, all the transmitted plane waves that reach the metallic interface undergo reflection with the coefficient $r_{23}=-1$. These waves subsequently undergo reflections and transmissions at the interface of the substrate and air with coefficients ${\tilde{r}}_{21}=r_{21}e^{i\phi_{21}}$ and ${\tilde{t}}_{21}=t_{21}e^{i\theta_{21}}$, as illustrated in Figure 9a. The total reflection can be calculated by superposing these multiple reflections.

(5) $$\tilde{r}={\tilde{r}}_{12}-\frac{{\tilde{t}}_{12}{\tilde{r}}_{21}e^{i2\beta }}{1+{\tilde{r}}_{21}e^{i2\beta }}$$

**Figure 10:**
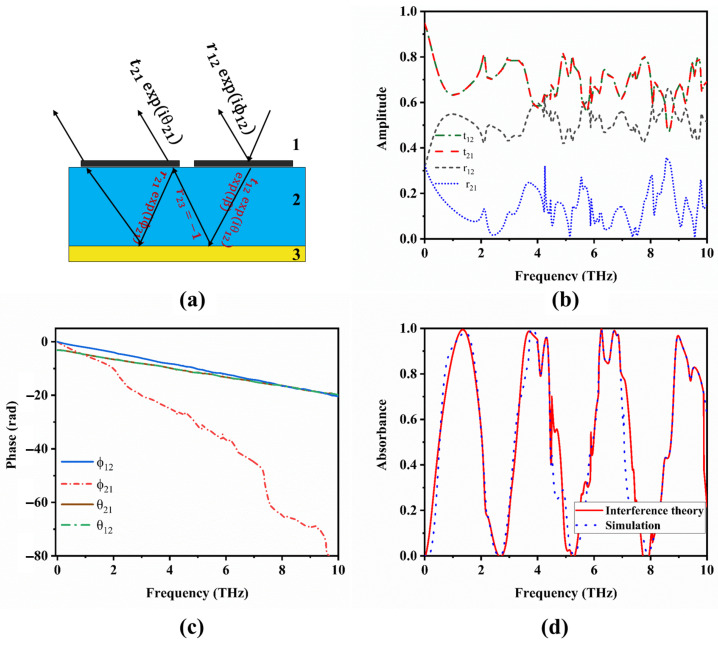
(**a**) A multi-reflection interference model of the absorber. (**b**) Amplitude of the reflection and transmission coefficients obtained from the absorber unit cell without a gold reflector. (**c**) Phase of the reflection and transmission coefficients obtained from the absorber unit cell without a gold reflector. (**d**) Comparison of absorption spectra obtained from the interference theory and simulation when the VO_2_ layer is in the conducting state.

The complex propagation phase $\beta =\sqrt{\varepsilon_{d}}k_{0}h$, where $\varepsilon_{d}$ is the permittivity of the substrate, *h* is the thickness of the substrate, and $k_{0}$ is the wave number. The absorption can be calculated as $A\left(\omega \right)=1-|\tilde{r}{\left(\omega \right)|}^{2}$. Figure 10b,c demonstrate the obtained reflection and transmission coefficients, as well as the corresponding phase, respectively, through simulations of the proposed metamaterial absorber without the metallic plane. The comparison between theoretical calculations and CST simulations is illustrated in Figure 10d. Significantly, the theoretical results align well with the simulation results, indicating a favorable agreement between the two.

**Figure 11:**
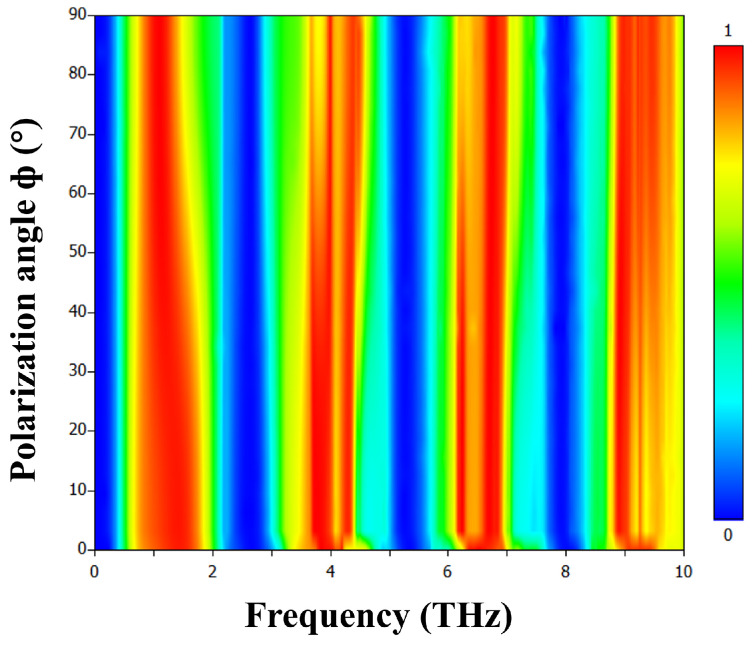
The simulation results present the absorptivity of the proposed metamaterial as a function of frequency and polarization angles, when the VO_2_ layer is in the conducting state at 344 K.

The polarization insensitivity of our tunable terahertz metamaterial multiband absorber was thoroughly investigated to assess its performance under different incident polarization angles. Figure 11 displays the absorptance as a function of the variation in polarization angle. The results demonstrate that the absorption bandwidth of the first peak becomes narrower at higher polarization angles. This phenomenon is attributed to the outer split ring, as shown in Figure 4e, which has a small impact on the absorption bandwidth. Overall, the performance of the absorber remains unaffected by changes in the polarization angle under normal incidence. This occurs due to the transverse field of incident electromagnetic waves, which can typically split into two polarized components orthogonal to each other. In our scenario, the structure exhibits orthogonal symmetry in the x-y plane, thereby equally interacting with each field component. Consequently, it displays insensitivity to electromagnetic waves with varying polarization angle. The proposed design ensures that the absorber retains its performance regardless of the rotation of the polarization angle.

**Figure 12:**
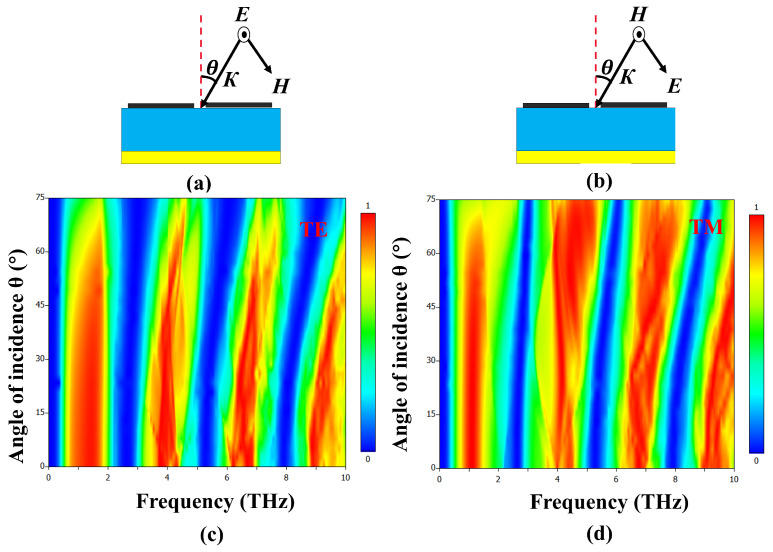
(**a**) Schematic illustration of absorber with changing incident angle θ for TE and (**b**) TM Polarization. The simulation results present the absorptivity of the proposed metamaterial as a function of frequency and angle of incidence for (**c**) TE and (**d**) TM polarizations, respectively, while the VO_2_ layer is in the conducting state at 344 K.

We explore the impact of the incident angle on our metamaterial absorber’s performance for both transverse electric (TE) and transverse magnetic (TM) polarizations in Figure 12c,d. Under TE polarization, the absorption bands remained above 90% up to a $30^{\circ }$ incident angle, but exhibited blue shifts beyond, indicating a change in response. The absorber maintained over 80% absorption up to $65^{\circ }$, with gradual decreases due to impedance mismatch as the incident angle increased. In contrast, TM polarization showed increased absorption with a rising incident angle, attributed to the enhanced vertical electric field component ($E_{Z}$). Both polarizations displayed resonant frequency blue shifts, attributed to Bragg scattering affecting higher order Fabry–Pérot resonant modes as changing incident angles disrupted electromagnetic wave propagation within the absorber.

**Figure 13:**
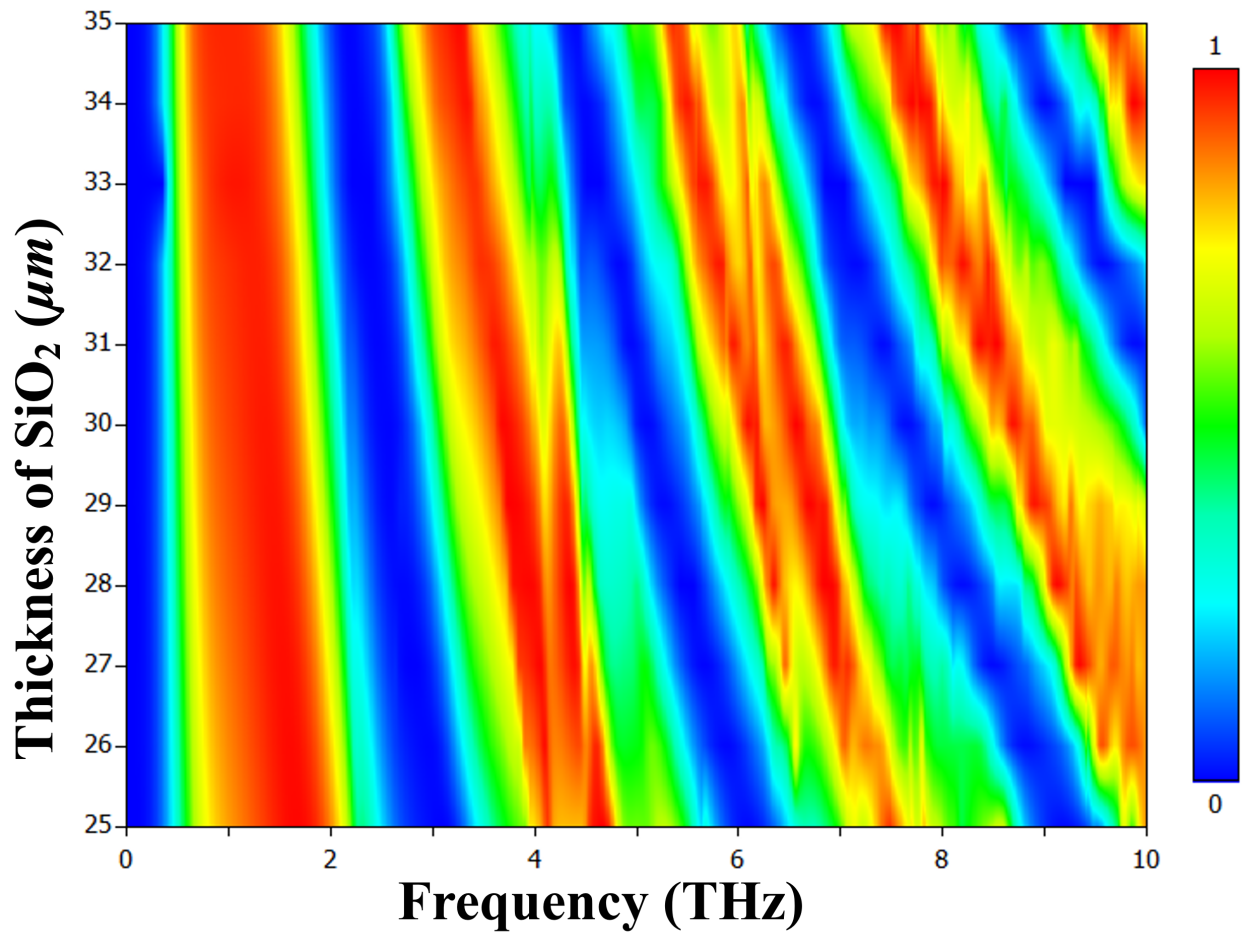
Absorption behavior as a function of frequency and thickness of SiO_2_, while all other parameters remain unchanged, and VO_2_ is in the conducting state at 344 K.

We investigated the impact of substrate thickness on our tunable terahertz metamaterial multiband absorber, as shown in Figure 13. As the dielectric substrate thickness increased from 25 to 35 μm, absorption peaks exhibited a red shift. This shift, attributed to phase propagation effects, resulted from the thicker substrate introducing a more prominent phase delay. Precise control of substrate thickness allows effective adjustment of absorption peak frequencies. Optimal performance was achieved with a 29 μm substrate, achieving maximum absorption efficiency due to surface resistance matching the impedance of the free space for all six peaks.

**Figure 14:**
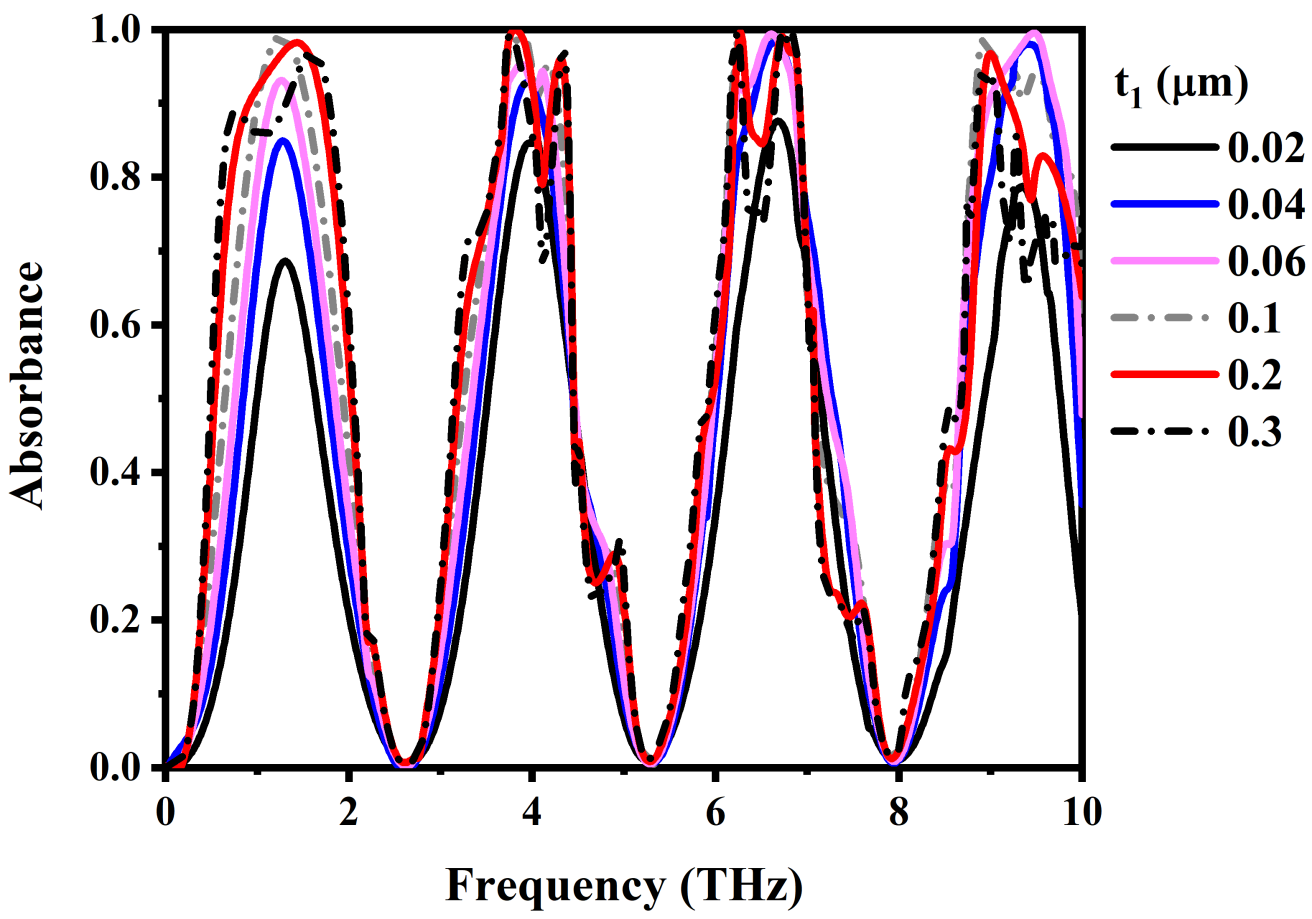
Calculated absorption spectra of the metamaterial absorber with different thicknesses of top VO_2_ resonators.

Figure 14 illustrates our investigation into the impact of VO_2_ thickness on absorption performance. A clear trend emerges as the VO_2_ thickness varies: absorption increases with thicker VO_2_ layers. This effect is attributed to increased interaction between incident terahertz waves and the VO_2_ layer, enhancing electromagnetic energy absorption. The metamaterial reaches peak absorption performance at a 0.2 μm VO_2_ thickness, demonstrating the highest absorption efficiency for incident terahertz waves. However, beyond this optimal thickness, absorptivity gradually decreases due to impedance mismatch with the free space at larger VO_2_ thicknesses.

## 4. Conclusions

In conclusion, we have successfully demonstrated a dynamic six-band terahertz metamaterial absorber based on VO_2_, as validated through theoretical and numerical analyses. In the conducting state of VO_2_, we attained six perfect absorption peaks, each with absorptances of 98.2%, 99.9%, 96%, 99.7%, 99%, and 96.4%. This dynamic tunability, ranging from 4% to 100%, was realized by the insulator-to-metallic transition of VO_2_. The absorption mechanism was thoroughly explained using interference theory, and our simulation results closely aligned with the theoretical results. Notably, multiple absorption peaks with a wide bandwidth cover 0.1 to 10 THz and maintain consistency under varying incident polarization angles. The simplicity of the design enhances its practicality for terahertz applications.

## Figures and Tables

**Table 1:** Performance comparison of this work with previously reported multiple-band absorbers.

Frequency (THz)	Number of Peaks	Average Absorption Rate (%)	Tunable Range (%)	Ref.
0.1–2	1	>90	20–99	[40]
5–8	2	99	36.2–99	[41]
1–9	3	99	30.4–99	[42]
0.1–12	3	>90	20–90	[38]
2–5	4	>97	–	[43]
6–10	4	>88	–	[44]
2.5–5.5	4	>96	–	[37]
0.1–9.5	5	>98	7–99	[45]
0.1–3	5	>98	–	[46]
0.1–10	6	>98	4–99.9	This work

## Data Availability

Data are contained within the article.
